# Spiky Gold Nanoparticles for the Photothermal Eradication of Colon Cancer Cells

**DOI:** 10.3390/nano11061608

**Published:** 2021-06-18

**Authors:** Paolo Emidio Costantini, Matteo Di Giosia, Luca Ulfo, Annapaola Petrosino, Roberto Saporetti, Carmela Fimognari, Pier Paolo Pompa, Alberto Danielli, Eleonora Turrini, Luca Boselli, Matteo Calvaresi

**Affiliations:** 1 Dipartimento di Farmacia e Biotecnologie, Alma Mater Studiorum—Università di Bologna, Via Francesco Selmi 3, 40126 Bologna, Italy; paolo.costantini4@unibo.it (P.E.C.); luca.ulfo2@unibo.it (L.U.); annapaola.petrosino2@unibo.it (A.P.); alberto.danielli@unibo.it (A.D.); 2 Dipartimento di Chimica “Giacomo Ciamician”, Alma Mater Studiorum—Università di Bologna, Via Francesco Selmi 2, 40126 Bologna, Italy; matteo.digiosia2@unibo.it (M.D.G.); roberto.saporetti2@unibo.it (R.S.); 3 Dipartimento di Scienze per la Qualità della Vita, Alma Mater Studiorum—Università di Bologna, Corso d’Augusto 237, 47921 Rimini, Italy; carmela.fimognari@unibo.it; 4 Nanobiointeractions and Nanodiagnostics, Istituto Italiano di Tecnologia, Via Morego 30, 16163 Genova, Italy; pierpaolo.pompa@iit.it

**Keywords:** photothermal therapy, gold nanoparticles, spiky nanoparticles, phototheranostics, colon cancer cells, NIR triggering

## Abstract

Colorectal cancer (CRC) is a widespread and lethal disease. Relapses of the disease and metastasis are very common in instances of CRC, so adjuvant therapies have a crucial role in its treatment. Systemic toxic effects and the development of resistance during therapy limit the long-term efficacy of existing adjuvant therapeutic approaches. Consequently, the search for alternative strategies is necessary. Photothermal therapy (PTT) represents an innovative treatment for cancer with great potential. Here, we synthesize branched gold nanoparticles (BGNPs) as attractive agents for the photothermal eradication of colon cancer cells. By controlling the NP growth process, large absorption in the first NIR biological window was obtained. The FBS dispersed BGNPs are stable in physiological-like environments and show an extremely efficient light-to-heat conversion capability when irradiated with an 808-nm laser. Sequential cycles of heating and cooling do not affect the BGNP stability. The uptake of BGNPs in colon cancer cells was confirmed using flow cytometry and confocal microscopy, exploiting their intrinsic optical properties. In dark conditions, BGNPs are fully biocompatible and do not compromise cell viability, while an almost complete eradication of colon cancer cells was observed upon incubation with BGNPs and irradiation with an 808-nm laser source. The PTT treatment is characterized by an extremely rapid onset of action that leads to cell membrane rupture by induced hyperthermia, which is the trigger that promotes cancer cell death.

## 1. Introduction

Colorectal cancer (CRC) is one of the major causes of cancer-related death [1]. Nearly two million new cases and about one million deaths are expected each year worldwide, with an increasing trend in CRC incidence, especially in more economically developed countries [1].

CRC is commonly treated by surgery; however, up to half of patients diagnosed with early-stage CRC experience recurrent disease after a surgical resection and may also develop a metastatic disease. As such, neoadjuvant and adjuvant therapies have a crucial role against CRC [2]. These include chemotherapy, radiotherapy, interventional therapy, and biotherapy [2,3]. Unfortunately, systemic toxic effects, which impair the patient quality of life, and the development of resistance during therapy limit the long-term efficacy of these therapeutic approaches, especially in metastatic cases. A search for alternatives is both timely and necessary in this case.

Thermal ablation and laser-induced thermotherapy are techniques that potentially address these issues [4,5]. These are localized physical treatments that use hyperthermia to damage and kill cancers cells and tumorous tissues and that do not develop resistance in cells [5]. Clinical trials are in progress to evaluate their safety and efficacy and additionally the treatment of metastatic CRC [6].

Similar to the heat-mediated cytotoxicity observed in thermal ablation and in laser-induced thermotherapy, photothermal therapy (PTT) represents an innovative treatment for cancer with great potential [7,8]. The procedure for PTT is based on accumulation of photosensitive molecules/nanoparticles in cancer cells, followed by light irradiation of the target tissue [9]. The irradiation with the appropriate wavelength (usually near-infrared (NIR) light) promotes photosensitizer activation from the ground state to any of their excited states. When they relax back to the ground state via non-radiative de-excitation, the energy dissipation causes a localized release of heat that causes severe damage to nearby cells and tissues [9]. In PTT, the intracellular temperature of cancer cells easily exceeds 50 °C, resulting in rapid cell death [9]. Compared to the traditional treatment methods, PTT has significant advantages including the use of soft and penetrating irradiation sources to activate the photothermal agent (NIR light) and lower collateral damage to healthy tissues because it is possible to focus the irradiation at the desired (localized) site of action.

Gold nanoparticles (GNPs), characterized by a very high light-to-heat conversion efficiency, are among the most important photothermal agents for PTT [9,10] and have shown remarkable results in recent years. Examples include cancer treatments (solid tumor ablation) reaching the clinical trial stage [11], suggesting promise for future applications [12,13,14]. The unique size and shape-dependent optical properties of GNPs, based on the localized surface plasmon resonance (LSPR) phenomenon, allow for tunable and intense absorption cross-sections and a consequent photothermal conversion ability. According to their size, GNPs present an extinction coefficient up to five order of magnitude larger than other molecular dyes commonly employed in PTT [15,16,17]. Among other shapes, anisotropic GNPs exist in rod-like, [18,19] prism-like, [19,20,21] and more recently branched (spiky) nanostructures [22,23,24,25], whose geometrical features can be controlled to obtain the LSPR in the near-infrared (NIR) spectrum and appear to be particularly promising due to superior light tissue penetration of NIR light [26].

Branched GNPs (BGNPs) present several advantages. These advantages include the lack of a need to involve highly toxic reagents in the synthesis process (i.e., CTAB, commonly employed for rods), and their complex nanostructure discloses a wider choice of shape-dependent biological and optical properties, which can be carefully tailored by controlling the NPs growth process to obtain the desired average length, width, and tip density, as well as the proper dimension of the central core [27,28,29,30]. Changing these parameters not only leads to a spectral shift in the LSPR but also to the modification of their absorption efficiency. While tuning the tip length (and the core-to-tip size ratio) allows modulating the absorption wavelength, the tip density, and the core size mainly impact the LSPR intensity. Here, BGNPs were designed for effective absorbance of NIR light, which is an attractive energy source because human tissues and blood are minimally absorptive in these wavelengths. NIR lasers and fiber optics, which represent minimally invasive and versatile energy delivery systems, are already commercially available, allowing an easy translation to the clinic of NIR based PTT. In the NIR region, two biological windows, i.e., spectral ranges where tissues are partially transparent due to a simultaneous reduction in both absorption and scattering, can be defined. The first biological window (I-BW) extends from 650 nm to 950 nm and corresponds to the spectral range delimited by the absorption of hemoglobin and water. The second biological window (II-BW) extends from 1000 nm to 1350 nm and it is limited by water absorption bands. The I-BW region is characterized by a negligible absorption from tissue and the photothermal agents represent the sole heating sources. In the II-BW window, water absorbs in the whole range, generating background heating upon irradiation, leading to a reduced PPT selectivity and efficiency.

In this work, we design and synthesize highly photostable BGNPs that can be strongly absorbed in the first biological window (I-BW). We develop protocols to ensure the colloidal stability of the BGNPs under physiological conditions and evaluate the BGNP photothermal conversion ability upon irradiation with an 808-nm laser light source. The applicative potential of BGNPs is tested in vitro against a well-established colon cancer cell line, using in vivo-like protein concentration conditions. Hereafter, the biocompatibility, cellular uptake, and ability of the BGNPs to eradicate colon cancer cells following PTT treatment are described.

## 2. Materials and Methods

### 2.1. Materials

All chemicals and reagents employed were of the highest technical grade available and stored following the vendor recommendations. Hydrogen tetrachloroaurate(III) hydrate (≥99.9%, Alfa Aesar, Haverhill, MA, USA, 42803), trisodium citrate trihydrate ReagentPlus^®^ (≥99%, SigmaAldrich, St. Louis, MO, USA, 25114), hydroquinone (SigmaAldrich, 605970), and O-(2-carboxyethyl)-O′-(2-mercaptoethyl) heptaethylene glycol (SigmaAldrich, 672688) were used. A PVC calibration standard at 483 nm (PVC000476) was purchased from Analytik Ltd. (Cambridge, UK).

### 2.2. Synthesis and Characterization of BGNPs 

The BGNP colloidal suspension was prepared by slightly modifying previously reported methods [27,31,32]. Briefly, 15-nm gold seeds were first synthesized by adding 4.5 mL of trisodium citrate (34 mM, 0.15 mmol) to 150 mL of HAuCl_4_⋅3H_2_O (0.038 mmol, 0.25 mM) in a boiling solution. After 30 min, the mixture was let cooled to RT, stirred overnight, and filtered through 0.2-µm syringe filters.

Then, 120 µL of HAuCl_4_⋅3H_2_O (95 mM), 72 µL of the prepared seeds, 280 µL of trisodium citrate (34 mM), and 280 µL of hydroquinone (140 mM) were subsequently added to 96 mL of ultraclean deionized H_2_O under vigorous stirring. After 2 min, 30 µL of O-(2-carboxyethyl)-O′-(2-mercaptoethyl) heptaethylene glycol was added to the colloidal suspension and the mixture was stirred for 6 h. The pegylated BGNPs were finally filtered through 0.4-µm syringe filters and purified from the free ligands by several washing cycles using centrifugal filters.

### 2.3. UV–Vis–NIR Absorption Spectroscopy 

BGNP absorption spectra were examined with a Varian Cary5000 using a 1 cm path length with Hellma quartz cells, measuring in the 400–1200 nm range. Stability tests in serum were performed with a Thermo Fisher NanoDrop^®^ (350–900 nm range) instrument using small-volume PMMA disposable cuvettes (Sarstedt, Nümbrecht, Germany). The samples were diluted prior to analysis to obtain an absorbance of ≤1.

### 2.4. Dynamic Light Scattering (DLS) Analysis 

BGNP sample analysis was performed using a Zetasizer Nano Range (Malvern, Worcestershire, UK) instrument and the reported values are an average of three independent measurements (each consisting of an accumulation of 11 runs).

### 2.5. Differential Centrifugal Sedimentation (DCS) Analysis 

The analysis was carried out using the CPS DC24000 UHR ultrahigh resolution particle analyzer (CPS Instrument Inc., Prairieville, LA, USA). DCS measurements were performed using 8–24% sucrose density gradient in ultraclean deionized water (or in PBS when measuring BGNPs stability in FBS) with a disc speed of 18,000 rpm. PVC standard particles (0.483 µm, Analytik Ltd., Jena, Germany) were employed to calibrate the instrument before each sample measurement.

### 2.6. Transmission Electron Microscopy (TEM) Analysis 

The samples were prepared by drop casting 1 µL of a BGNP suspension on a formvar-coated copper grid cleaned with oxygen plasma (200 mesh, Ted Pella, Redding, CA, USA) and were left to dry in air for 2 h. A JEOL JEM 1400 microscope (JEOL, Tokyo, Japan) operating at 120 kV was employed for imaging. The images were analyzed using ImageJ to estimate the mean BGNP diameter (average of the longest tip-to-tip distance).

### 2.7. Inductively Coupled Plasma (ICP-OES) Elemental Analysis 

ICP elemental analysis was performed by inductively coupled plasma optical emission spectroscopy (ICP-OES) using a ThermoScientific (Waltham, MA, USA) iCAP 6300 DUO ICP-OES spectrometer. Chemical analyses by ICP-OES are affected by a systematic error of 5%. Furthermore, 30–50 µL of a BGNP sample was dissolved overnight in 1 mL of aqua regia and then diluted to 10 mL with ultraclean deionized water before analysis.

### 2.8. BGNPs-Biomolecular Corona Preparation 

Next, 50 μL of BGNP stock solution (2.5 mM Au^0^) was diluted with 250 μL of fetal bovine serum (FBS) and gently mixed by pipetting. Then, 200 μL of the RPMI cell medium was added to the mixture, obtaining a final concentration of 0.25 mM Au^0^ of BGNPs in 50% fetal bovine serum (FBS). Following this, 1% L-glutamine at 200 mM and 1% penicillin/streptomycin solution at 100 U/mL were added for in vitro experiments. The mixture was then incubated at 37 °C under continuous shaking (700 rpm) for 1 h (ThermoMixer HC, S8012-0000; STARLAB, Hamburg, Germany).

### 2.9. Cell Culture

The authenticated colorectal cancer cells (DLD1) were obtained from LGC Standards (Teddington, Middlesex, United Kingdom), grown in adhesion, and propagated in a RPMI 1640 medium supplemented with 10% heat-inactivated fetal bovine serum (FBS), 1% L-glutamine 200 mM, and 1% penicillin/streptomycin solution 100 U/mL (all purchased by Euroclone, Pero, Italy). Cells were maintained at 37 °C in a humified atmosphere containing 5% CO_2_.

### 2.10. Instrumental Setup for Photothermal Measurements 

The photothermal performances of BGNPs were evaluated by measuring the temperature increase during NIR laser irradiation on 96-well plate. A fully automated experimental setup in collaboration with Crisel Instruments (Rome, Italy), integrating a (i) NIR laser source, (ii) XY micropositioning stage, and (iii) thermal camera, was used to perform the treatment.

#### 2.10.1. NIR Laser Source 

CW fiber-coupled infrared diode lasers (MDL-F-808; CNI Optoelectronics; Changchun, China) with a nominal power of 2.2 W and emission wavelength of 808 nm were used. A multimode fiber with a core diameter of 400 μm was used to couple the laser with a collimating spherical lens (FOC-01-B, CNI Optoelectronics; Changchun, China), producing a spot size of 6.5 mm at a distance of 10 cm, matching the single well diameter (for 96-well plate). The laser irradiation comes from the top and is perpendicular to the multiwell plate.

#### 2.10.2. XY Micropositioning Stage 

The positioning of the plate under the laser spot was performed using the XY micropositioning OptiScan^®^ stage (Prior Scientific, Cambridge, UK), including the XY stage, controller, joystick, and plate holder. The stage was remotely controlled with the Micromanager control software.

#### 2.10.3. Thermal Camera 

Real-time temperature changes were recorded by thermal images acquired with an Optris Xi 400 camera (Optris, Berlin, Germany) coupled with a 18° × 14° lens (f = 20 mm) at a framerate of 27 Hz. Data acquisition and analysis were performed with Optrix PIX Connect software.

### 2.11. Photothermal Treatment in Phantom System 

The light to heat conversion performances of the BGNPs were evaluated using the abovementioned integrated setup. Volumes of 200 μL of the different solutions was used to carry out all the PTT measurements. Different concentrations of BGNPs (0.25, 0.1, 0.05, 0.025 mM Au^0^) were prepared by diluting the stock solution with milliQ water.

### 2.12. Cell Treatment 

About 15,000 cells were seeded in each well in a 96-well plate. After overnight incubation, cells were treated with FBS-dispersed BGNPs for 24 h. Cells were washed twice with RPMI supplemented with 50% FBS in order to remove free BGNPs, and then were irradiated with an 808 nm NIR laser at 3.3 and 6.6 W cm^−2^ for 1 or 3 min. At the end of irradiation, the medium was removed and cells were incubated for 3, 6, or 24 h according to experimental exigences for the analysis.

### 2.13. BGNP Uptake by Flow Cytometry 

After 24 h of BGNP exposure, cells were trypsinized, centrifuged at 1200 rpm for 5 min, and resuspended with PBS for the uptake analysis via flow cytometry. A side scattering setting associated with a 488-nm excitation laser was used to detect BGNPs uptake [33,34]. The median fold-increase was used to quantify the SSC of BGNP-treated cells compared to not treated cells. All the flow cytometric analyses were performed using an EasyCyte 6-2L (Guava Technologies, Merck, Darmstadt, Germany) instrument. At least 10,000 events were evaluated for each analyzed sample.

### 2.14. BGNP Uptake by Confocal Microscopy 

Following the cell treatment, cells were fixed on 12-mm glass coverslips with 4% paraformaldehyde (PFA) in PBS for 15 min at RT, then washed three times with PBS. The coverslips were mounted with an antifade mounting medium (vectashield H-1000, Vector Laboratories, Burlingame, CA, USA) on a glass microscopy slide. Images were acquired by a confocal microscope (Leica SP8 TCS, Leica Microsystems GmbH, Wetzlar, Germany) with 63× and 100× oil immersion objectives. Z-stacks were acquired by using a 1024 × 1024 scan format and 400 msec speed.

### 2.15. Cell Viability and Analysis of Cell Death Mechanism 

To assess DLD1 viability, a MTT test was performed. Briefly, after cell treatment with BGNPs and 24 h after irradiation, the media were removed from each well and cells were incubated with a 0.5 mg/mL MTT solution for 90 min at 37 °C with 5% CO_2_. At the end of the incubation, the MTT solution was removed and formazan salts were dissolved in 100 µL of DMSO. The absorbance was measured at 570 nm using an EnSpire multimode microplate reader (Perkin Elmer, Waltham, MA, USA). To investigate if the recorded cytotoxic effects were triggered by regulated mechanisms, such as the apoptotic cell death, the Guava Nexin Reagent (Merck, Dramstadt, Germany) was used while containing 7-aminoactinomycin (7-AAD) and annexin V-phycoerythrin. Early events in regulated cell death are characterized by the exposure of phosphatidylserine on the cell membrane, whose integrity remains preserved. Exposed phosphatidylserine is detected by the binding protein annexin V. On the contrary, necrotic cells suffer from the loss of membrane integrity, resulting in being permeable to the dye 7-AAD, which is a DNA intercalator. Accordingly, by using the Guava Nexin Reagent it is possible to distinguish three cell populations, i.e., (i) living cells (annexin V −/7-AAD −), (ii) cells undergoing early phases of regulated cell death (annexin V +/7-AAD −), (iii) and necrotic cells (annexin V +/7-AAD +). Cell viability was analyzed at 3, 6, and 24 h post-irradiation. According to manufacturer instructions, 2 × 10^4^ cells were stained with the reagent for 20 min in the dark at room temperature and analyzed via flow cytometry.

### 2.16. Statistical Analyses 

Results are expressed as the mean ± SEM of at least three independent experiments. The analysis of variance for reported measures and Bonferroni as posttest were used. The statistical software GraphPad InStat 5.0 version (GraphPad Prism, San Diego, CA, USA) was used. In this work, *p* < 0.05 was considered significant.

## 3. Results and Discussion

### 3.1. Synthesis and Characterization of BGNPs

A BGNP colloidal suspension was prepared by seed-mediated synthesis [31,32] (see experimental section) to guarantee good shape control [27,30,35,36], despite the irregular (non-geometrical) shape. This aspect is crucial when aiming for a biological application, since different shapes can lead to different biomolecular corona, uptake, biodistribution, and immune response characteristics [27,37,38,39,40]. The BGNPs were functionalized with O-(2-carboxyethyl)-O′-(2-mercaptoethyl) heptaethylene glycol (HS-PEG_7_-COOH) to gain better colloidal stability and strong surface anchoring, which are essential to maintain and stabilize the shape. The short length of the thiol ligand was selected to allow high-density coating, potentially providing better resistance of the nanostructure (and therefore of the optical properties) to thermal annealing and laser irradiation [41]. Furthermore, compared to the bare NPs, the negatively charged PEG ligand coating helps reduce the strength of protein interactions with the surface [42,43], while a positive charge can lead to stronger interactions with proteins [44].

**Figure 1:**
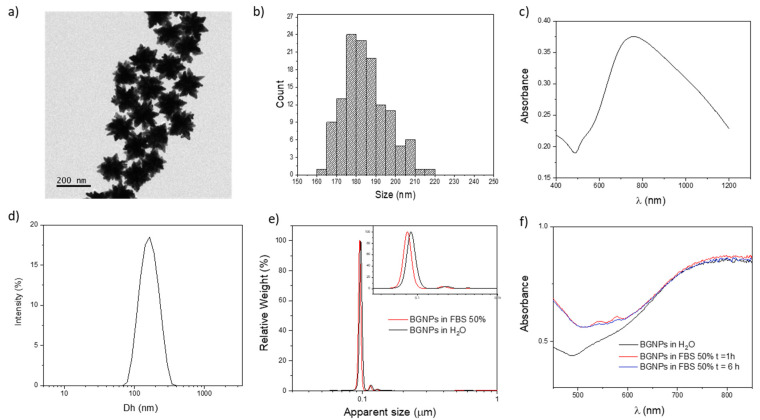
Characterization of BGNPs. (**a**) TEM micrograph. (**b**) TEM BGNP size distribution. (**c**) Vis–NIR absorption spectrum in H_2_O. (**d**) DLS analysis (Dh = 164 nm, PDI = 0.06). (**e**) DCS analysis of BGNP in H_2_O and cell culture media FBS 50% (apparent sizes of 100 nm and 97 nm respectively). (**f**) Absorption spectra related to the stability test of BGNPs in biological media: BGNPs were incubated in 50% FBS (in PBS) at 37 °C for different times (1 and 6 h). In the case of anisotropy, DLS and DCS do not represent the actual particle diameter. Consequently, representative particle distributions are reported rather than the actual sizes because of the incorrect geometrical assumption/approximation involved in the measurements.

BGNPs were fully characterized (see Figure 1). TEM analysis showed a monodistributed sample with an average size (calculated measuring the particle longest tip-to-tip distance) of 180 ± 10 nm (Figure 1a,b); the nanostructures presented a large core with a multitude of tips, which are the main responsible for the characteristic NIR-LSPR band centered near 800 nm (Figure 1c). The DLS and DCS analyses confirmed the high quality of the prepared BGNPs and the monodisperse size distribution (Figure 1d,e). Only the apparent size of BGNPs could be obtained by DCS in this case, as the technique estimates the diameter considering the object analyzed as a solid sphere of a specific density. The technique is nevertheless suitable to spot the presence of multiple populations or aggregation. In addition, it allows performing direct analysis of the BGNPs in the biological media with no need for purification/isolation steps to remove the excess of proteins. The stability test of the BGNPs in cell culture media showed excellent stability of the sample, with only a slight shift towards smaller sizes due to protein adsorption (biomolecular corona), which leads to a minor total density of the particles (Figure 1e,f) [45]. Further tests in 50% FBS (the in vivo-like conditions employed in our in vitro studies) were performed by absorption spectroscopy, confirming the stability of BGNPs. The slight DCS shift (3 nm) and the absence of a significant shift in the LSPR suggested a limited strength of the NP–proteins interaction, as expected (see Figure 1f). Nevertheless, the observed protein corona formation can actually lead to improved colloidal stability and biocompatibility [46,47] and might still play an essential role in the particle interaction with cells (i.e., specific interactions with cell receptors and internalization) [48,49]. For this reason, to present a more realistic behavior of the BGNPs in the biological environment (different protein concentrations can lead to different biomolecular corona compositions), [50] the nanostructures were exposed to an in vivo like protein concentration (50% *v*/*v* of serum), which will also be employed for in vitro testing [51].

### 3.2. Photothermal Performances of BGNPs in Phantom System

**Figure 2:**
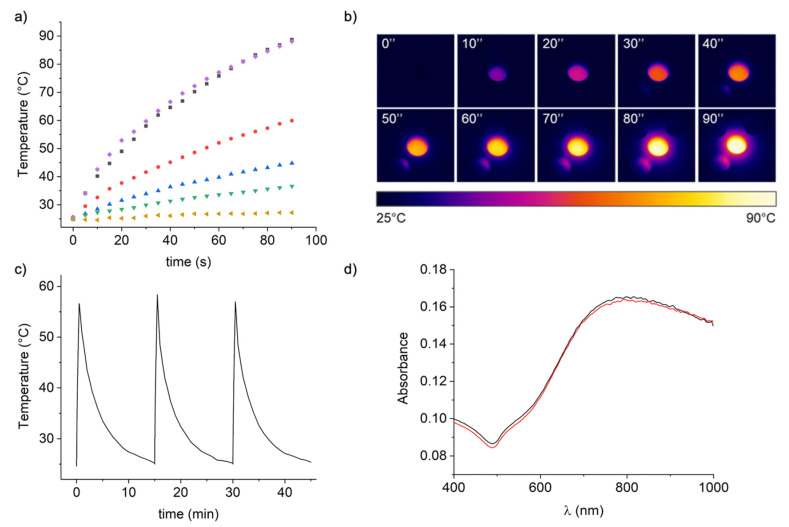
The photothermal effect of BGNPs in a phantom system. (**a**) Heating curves for different concentrations of BGNPs in water (0.25 mM Au^0^ in violet, 0.1 mM Au^0^ in red, 0.05 mM Au^0^ in blue, 0.025 mM Au^0^ in green, H_2_O in yellow) and the BGNP biomolecular corona in water (0.25 mM Au^0^ in black) during 808 nm NIR laser irradiation (6.6 W cm^−2^). (**b**) Thermal imaging of the solution containing BGNPs (0.25 mM Au^0^) for different times. (**c**) Temperature change of the solution containing BGNPs (0.25 mM Au^0^), showing three laser on/off cycles of 808 NIR laser (6.6 W cm^−2^). The sample was heated for 30 s, then the laser was switched off for 15 min and the solution was left to cool. (**d**) Vis–NIR spectra of the BGNPs before (black) and after (red) laser irradiation/heating.

To explore the photothermal conversion efficiency of the BGNPs, we monitored the temperature of water solutions containing different concentrations of BGNPs during laser irradiation. The photothermal heating curves take the transferred heat from the BGNP to the medium into account. As such, the medium temperature only allows for an indirect view of the heat generated locally. In fact, the nanoparticles themselves may have much higher temperatures in their close proximity. Temperature differences of 70–90 °C were observed over distances of ∼100 nm [52]. This aspect is crucial in biological experiments, where cells are sensitive to the local temperature of the nanoparticles, rather than medium temperature. The solutions were exposed to an 808-nm laser light source at a fixed power density (6.6 W cm^−2^) for 90 s. The photothermal heating curves (Figure 2a), measured by an IR thermal camera (Figure 2b), showed a concentration-dependent photothermal effect, with the highest temperature increment of the solution with 0.25 mM Au^0^ up to 65 °C (from 25 °C to 90 °C). The temperature increased proportionally with the increase of BGNPs concentration. In contrast, a negligible heating of only 2.2 °C was observed for water without BGNPs at the same exposure conditions. To provide a quantitative heating efficiency of our BGNPs comparing the results to similar gold nanoparticles presented in literature, the molar rate of heat transfer (Equation (1)) was calculated as proposed by Kuttner et al. [53].

(1) $$\frac{\Delta Q}{c_{\mathrm{Au}}}=\frac{\left(Q_{\mathrm{sample}}-Q_{\mathrm{medium}}\right)}{c_{\mathrm{Au}}}$$

The delivered thermal energy Δ*Q* = (*Q*_sample_ − *Q*_medium_) was calculated following the method described by Roper et al. [54] and Quintanilla et al. [55] (see Equations (S1)–(S4) and Figure S1 in Supplementary Materials), while the *c*_Au_ was calculated by inductively coupled plasma (ICP-OES) elemental analysis. The result of molar heat transfer rate is 0.53 W mM^−1^, which is a value similar to suitable gold nanoparticles in PTT, such as gold nanorods [53].

The interaction with proteins may affect the photophysical properties of photosensitizers, [56,57,58,59,60,61] so we evaluated the BGNP performances in a physiological-like conditions, investigating the possible effects of the biomolecular corona during laser the irradiation/heating process [44]. We repeated the irradiation experiment for the FBS-dispersed BGNPs, using the highest concentration of BGNPs (0.25 mM Au^0^ BGNPs). The results (Figure 2a, curves in violet and in black) are practically superimposable to the protein-free sample, indicating that the protein corona does not affect the photothermal behavior of the nanoparticles. It is known that some photothermal agents can degrade and eventually lose their photothermal properties during laser irradiation, in particular organic dyes such as cyanines and photobleach [62]. Other nanoparticles, like gold nanorods, are known to change their structure with laser absorption [63,64]. Thus, it is important to determine the photothermal stability of BGNPs to exclude heat-induced morphological changes and consequent LSPR shifts, which could prevent further cell death during in vitro laser treatment. As such, the BGNPs were subjected to multiple irradiation cycles (Figure 2c) and high-temperature treatment. A variation of 32 ± 1 °C (from 25 °C to 57 ± 1 °C) was obtained within 30 s and the light-to-heat conversion performances were maintained over the three cycles of heating and cooling performed, confirming the reproducibility of the photothermal response of the BGNPs (Figure 2c). To ensure the stability of the BGNPs after irradiation, the vis–NIR spectra were recorded before and after NIR laser irradiation (Figure 2d). The vis–NIR absorption spectrum of BGNPs remains unchanged after the three sequential cycles of heating and cooling, revealing that NIR irradiation does not affect the BGNPs colloidal stability and that there is no structural rearrangement of the gold nanoparticle due to laser irradiation/thermal heating. In fact, it is well-known that aggregation phenomena and the surface modification of BGNPs cause evident changes in their vis–NIR spectra. In addition, these results clearly confirm that BGNPs does not photodegrade during NIR treatment, as opposed to many organic dyes commonly used as photothermal agents. TEM analysis after irradiation were also performed (see Figure S2), confirming the thermostability of the nanostructures.

### 3.3. Uptake of BGNP in Colon Cancer Cells

**Figure 3:**
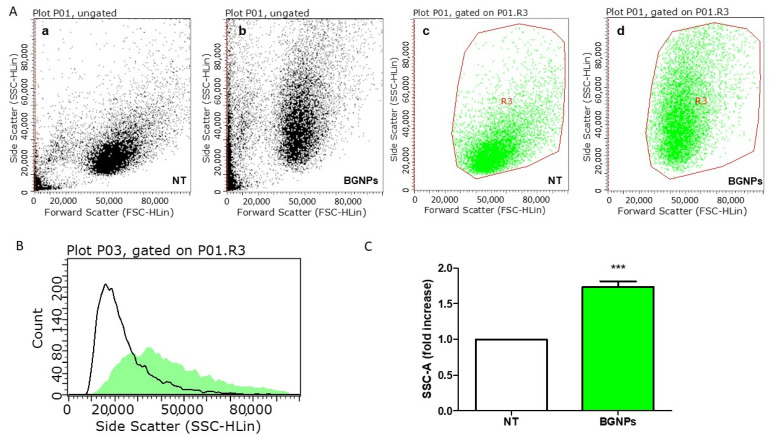
Flow-cytometric analysis of DLD1 after 24 h of BGNP exposure. (**A**) Representative gating plot of side scattering (SSC-A) versus forward scattering (FCS-A) of non-treated (NT) cells and BGNP-treated cells with all acquired events (**a**,**b**) and gated on living cells (R3) (**c**,**d**); (**B**) representative overlay of a side scattering histogram of cells exposed to BGNP compared to NT cells (dark line); (**C**) median relative fold-increase of SSC compared to NT cells of four independent experiments. *** *p* < 0.001 versus NT cells.

A representative colon cancer cell line, DLD1, was used to study the uptake of BGNPs. To better take into account the potential influence of bio-nano interactions and biomolecular corona on the BGNP cell uptake (which are protein concentration-dependent), in vitro experiments were performed using culture media supplemented with 50% of the serum to get closer to an in vivo-like scenario with regards to the protein concentration. A large excess of protein can influence the nature of the biomolecular corona and the interactions of BGNPs with the cell [65]. In fact, binding competition of the free proteins crowds the media and commonly leads to reduced uptake, especially for large NPs [66]. BGNPs pre-dispersed with 50% FBS were incubated with DLD1 cells for 24 h, and then the cellular uptake of the BGNPs was studied by flow cytometry. BGNPs are phototheranostic platforms [60,67] that allow both therapy (PTT) and label-free imaging. In fact, BGNPs can be used for optical imaging because of their capacity to absorb and scatter light in the visible and NIR regions. In particular, the LSPR responsible for the photothermal effects of BGNPs, also provides large scattering cross sections, allowing for convenient detection of the BGNPs by scattering-based detection methods. Flow cytometry can measure quantitatively intracellular GNPs by collecting the light scattering from a large population of living cells through efficient single-cell analysis [33]. In flow cytometry, there are two modes of scattering measurements: side scattering and forward scattering. The side scattering channel (SSC) is commonly used as an indication of the cell’s internal complexity or granularity. When nanoparticles are internalized by cells, the SSC intensity increases as a consequence of augmented intracellular complexity [33]. The (gated) side (SSC-A) and forward scatter (FSC-A) plots for DLD1 and DLD1 BGNPs-treated cells are reported in Figure 3A. Debris and death cells were excluded from the analysis based on morphology, thus gating the viable cells (R3) (Figure 3(Ac,Ad)). The cell granularity (SSC-A channel) of BGNPs-treated cells increased 1.74-fold compared to unexposed cells (Figure 3B,C), clearly indicating BGNP uptake. The same gating scheme was used for all the experiments.

**Figure 4:**
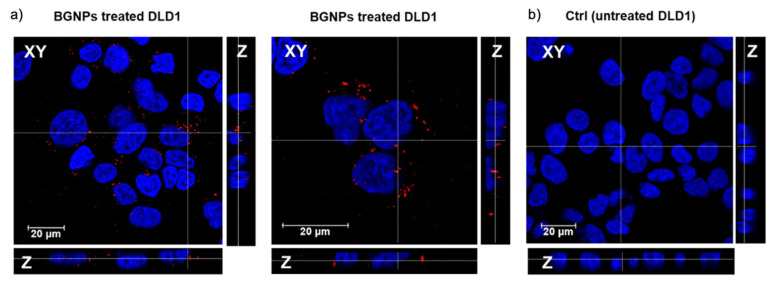
Confocal microscopy analysis. Representative images showing the XY planes and Z projections for (**a**,**b**) DLD1 cells after 24 h of BGNP exposure and (**b**) untreated DLD1 cell control. Nuclei are visualized by Hoechst staining (blue); BGNPs are visualized by reflected light (red). Bright filed XY transmission images are reported in Figure S3.

To corroborate the BGNP cell internalization, reflectance confocal imaging [68] was performed in order to directly exploit the optical properties of the nanomaterials (label-free approach), avoiding potential problems related to dye leaching and conjugations, which inevitably alter the surface chemistry of the NPs and potentially also their biological interactions [69]. Performing a Z-stack across the whole cell body allowed the observation of the presence of BGNPs inside the cell cytoplasms (red spots) while in the close proximity of the nuclei (see Figure 4), where they are likely to be accumulated in the lysosomes [22,70].

### 3.4. Efficacy of PTT Treatment in Colon Cancer Cells

**Figure 5:**
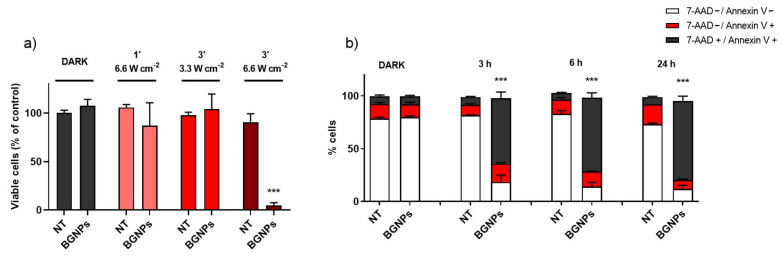
PTT treatment in colon cancer cells. (**a**) Percentage of viable cells (MTT) incubated with or without BGNPs, in dark condition or irradiated for different times and laser irradiance determined by MTT test. Percentage of viable cells is normalized on not treated (NT) cells in dark. (**b**) The percentage of living cells (white), cells undergoing programmed cell-death (red), and necrotic cells (black) after incubation or not with BGNPs in the dark or 3 h, 6 h, and 24 h after 3 min of irradiation at 6.6 W cm^−2^. Data are the mean values of at least three independent experiments. *** *p* < 0.001 versus non-treated (NT) cells.

To investigate the efficacy of BGNP-mediated PTT treatment on a cancer cell line, we incubated the DLD1 cells with 0.25 mM Au^0^ BGNPs that were pre-dispersed in FBS for 24 h. After washing to remove the BGNPs that were not taken up, the DLD1 cells were irradiated with a NIR laser for different irradiance times and intensities. After 24 h from the irradiation, cell viability was measured using the MTT test. One of main concerns related to nanoparticle-based treatments is their potential intrinsic toxicity. Despite the well-known biocompatibility of GNPs, it is crucial to perform case-by-case studies to exclude size/shape-depended cytotoxicity against this particular cell line. Therefore, MTT assays were performed, to confirm the biocompatibility of BGNPs in the absence of laser irradiation (Figure 5a). No cytotoxic effect was observed on BGNPs treated DLD1 cells in dark conditions. On the opposite, a remarkable decrease in cell viability (4.76% viable cells, see Figure 5a) was observed after 3 min of irradiation at 6.6 W cm^−2^. Interestingly, there was no cytotoxic effect when reducing the exposure time to 1 min or halving the laser irradiance to 3.3 W cm^−2^ while maintaining a period of 3 min of irradiation (Figure 5a).

These results let us hypothesize the presence of a threshold for PTT to achieve cell death. The level of this threshold is paramount to reducing PTT side effects. For example, photosensitivity represents a major side effect of photodynamic therapy (PDT), in which a patient remains photosensitive for several weeks after cessation of the treatment, because sunlight or bright lights may activate a non-controlled generation of ROS, induced by the non-eliminated photosensitizer. In our case, BGNPs only generate heat/phototoxicity in the presence of a controllable source of laser light. Direct sunlight (~0.1 W cm^−2^) or bright lights have irradiance intensities well below the threshold for BGNPs activation, thus they are not able to activate BGNP-dependent PTT. This aspect increases the control of the therapy such that PTT is activated only at the desired (localized) site of action, i.e., where the irradiation is focused, without collateral damage to surrounding tissues.

Typically, the application of PTT produces rapid temperature ramping, causing cellular death. Hyperthermia leads to cell membrane rupture, DNA damage and protein denaturation [13]. To discriminate the mechanisms of cell death, cells were counted for annexin V and 7-AAD staining in a flow cytometer, after 3 min of irradiation at 6.6 W cm^−2^, with or without FBS-dispersed BGNPs. No significant increase in programmed cell death events was recorded, nor after short (3 h, 6 h) or long (24 h) post-treatment times. In contrast, a significant increase in the fraction of necrotic cells was observed already after 3 h after irradiation in BGNP-treated cells when compared to non-treated cells (62% versus 7%, respectively) (Figure 5b). Necrotic events reached the highest percentage 24 h after irradiation (75%) (Figure 5b). These results indicate that under laser irradiation, BGNPs induce cellular necrosis as the main cell death mechanism, conceivably due to the direct effect of the thermal stress on the cells. Our findings agree with several previous studies that identify necrosis as the main in vitro cellular response to PTT [13].

## 4. Conclusions

In this work, we have synthesized branched gold nanoparticles (BGNPs) as attractive agents for the photothermal eradication of colon cancer cells. The optical properties of the BGNPs were carefully tailored for effective absorbance in the first biological NIR window, a wavelength region of the light characterized by an optimal tissue penetration.

The FBS dispersed BGNPs were stable in physiological-like environments and were irradiated with an 808 nm laser source. They show an extremely efficient light-to-heat conversion capability. Sequential cycles of heating and cooling did not affect the BGNP stability.

Exploiting the intrinsic optical imaging offered by BGNPs, the uptake of BGNPs in colon cancer cells was confirmed using flow cytometry and confocal microscopy. In dark conditions BGNPs were fully biocompatible, while, when irradiated, BGNP-mediated PTT triggered rapid (3 h) cell death characterized by cell membrane rupturing, as evidenced by the high proportion of necrotic cells. These results agree with previous studies that identified necrosis as the main in vitro cellular response to PTT, leading to cell membrane rupture, DNA damage and protein denaturation. The passive accumulation of GNPs within cancer tissues, mediated by the enhanced permeability and retention effect, together with the possibility to easily functionalize the gold surfaces with targeting ligands [71], paves the way to providing robust double-targeting therapy approaches. The latter could exploit the recognition ability of conjugated targeting moieties with the possibility to focus the triggering light radiation at the desired site of action, lowering the collateral damage to healthy tissues, thus working towards a clinical need of crucial importance for the treatment of colon cancer.

## Data Availability

The data presented in this study are available on request from the corresponding authors.

## Supplementary Materials

The following are available online at https://www.mdpi.com/article/10.3390/nano11061608/s1, Figure S1: Representative cooling curve of a dispersion of BGNPs with an exponential regression, Figure S2: Nanostructure thermal stability. TEM micrographs of BGNPs before and after laser irradiation, Figure S3: Confocal microscopy analysis of BGNPs uptake.
