# Supplementary material for: Spiky Gold Nanoparticles for the Photothermal Eradication of Colon Cancer Cells
Source: Nanomaterials (Basel). 2021 Jun 18;11(6):1608. doi: 10.3390/nano11061608 (PMC8233824; doi:10.3390/nano11061608)
Supplement: Supplementary file 1 [file nanomaterials-11-01608-s001.zip › nanomaterials-1240156-supplementary.pdf]

nanomaterials

MDPI

Supplementary Materials

Spiky Gold Nanoparticles for the Photothermal Eradication of Colon Cancer Cells

Paolo Emidio Costantini [1,†], Matteo Di Giosia [2,†], Luca Ulfo [1], Annapaola Petrosino [1], Roberto Saporetti [2], Carmela Fimognari [3], Pier Paolo Pompa [4], Alberto Danielli [1], Eleonora Turrini [3,*], Luca Boselli [4,*] and Matteo Calvaresi [2,*]

[1] Dipartimento di Farmacia e Biotecnologie, Alma Mater Studiorum—Università di Bologna, via Francesco Selmi 3, 40126 Bologna, Italy; paolo.costantini4@unibo.it (P.E.C.); luca.ulfo2@unibo.it (L.U.); annapaola.petrosino2@unibo.it (A.P.); alberto.danielli@unibo.it (A.D.)
[2] Dipartimento di Chimica "Giacomo Ciamician", Alma Mater Studiorum—Università di Bologna, Via Francesco Selmi 2, 40126 Bologna, Italy; matteo.digiosia2@unibo.it (M.D.G); roberto.saporetti2@unibo.it (R.S.)
[3] Dipartimento di Scienze per la Qualità della Vita, Alma Mater Studiorum—Università di Bologna, corso d'Augusto 237, 47921 Rimini, Italy; carmela.fimognari@unibo.it
[4] Nanobiointeractions and Nanodiagnostics, Istituto Italiano di Tecnologia, Via Morego 30, 16163 Genova, Italy; pierpaolo.pompa@iit.it
* Correspondence: eleonora.turrini@unibo.it (E.T.); luca.boselli@iit.it (L.B.); matteo.calvaresi3@unibo.it (M.C.)
† These authors equally contributed to this work.

The molar rate of heat transfer is defined as Kuttner et al. [1].

$$\frac{\Delta Q}{c_{\mathrm{Au}}} = \frac{(Q_{\mathrm{sample}} - Q_{\mathrm{medium}})}{c_{\mathrm{Au}}} \tag{S1}$$

ΔQ represents the heat power released by the nanoparticles and the solvent. At the illumination wavelength (808 nm) used in the irradiation experiments, water is very weakly absorbing (see Figure 2a) and thus Q_{medium} is negligible. Q_{sample} can be defined as:

$$Q_{\mathrm{sample}} = \frac{m\, C_{\mathrm{sample}}\, \Delta T}{\tau} \tag{S2}$$

where m is the mass of the solution, C_{sample} is the heat capacity of the sample (it can be approximate as water in the used experimental conditions), ΔT is the increment of the temperature of the sample, and τ is the cooling rate. [1,2] This last parameter quantifies the heat dissipation with the surroundings, which is accurately measured from the cooling curves. Accordingly, the experimental procedure to determine τ involves an irradiation of the sample, then the illumination is turned off to let the cooling cycle start.

Figure 2c in the main text shows representative cooling curves of the dispersion of BGNPs.

Using Equation (S3) [3] the fitting of the data (Figure S1), provided a value of τ of 200 s.

$$\ln \theta = -\frac{t}{\tau} \tag{S3}$$

Where θ is a dimensionless parameter [3] calculated as in the Equation (S4).

$$\theta = \frac{T_{\mathrm{amb}} - T}{T_{\mathrm{amb}} - T_{\mathrm{max}}} \tag{S4}$$

Where T_{amb} is the ambient value of temperature, T_{max} is the maximum value reached by the temperature and T is the instantaneous temperature measured.

Nanomaterials **2021**, *11*, 1608. https://doi.org/10.3390/nano11061608

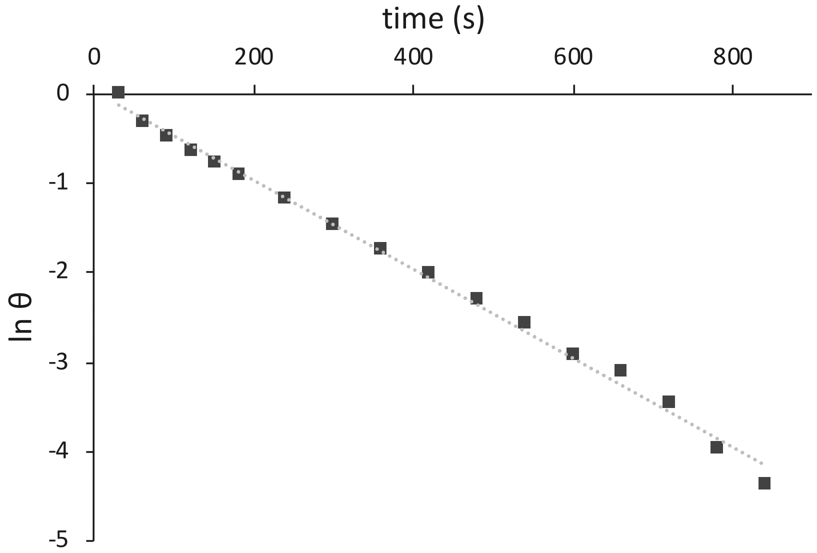

Figure S1. Representative cooling curve of a dispersion of BGNPs with an exponential regression.

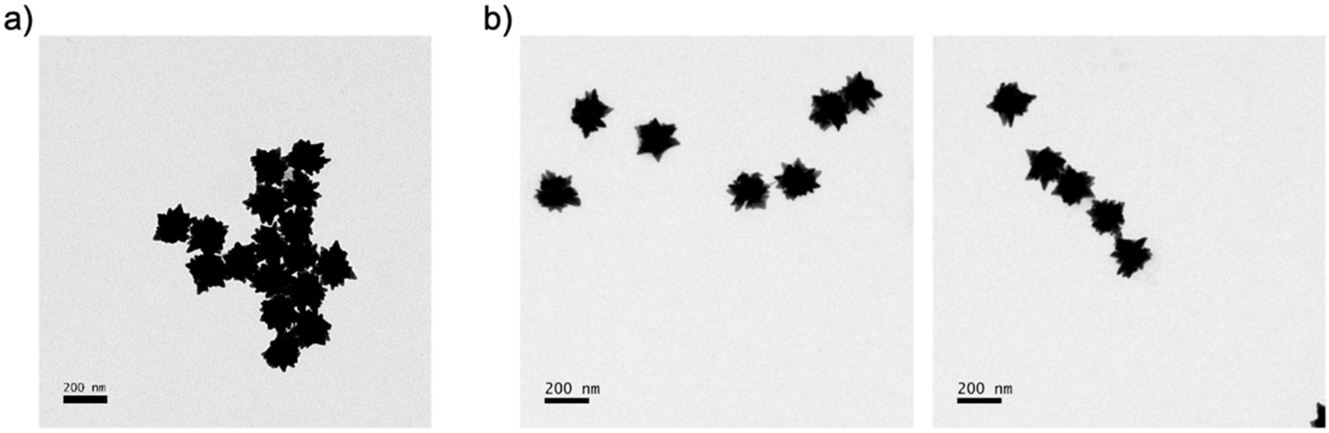

Figure S2. Nanostructure thermal stability. TEM micrographs of BGNPs **a**) before and **b**) after laser irradiation (the solution was exposed for 3 min to a 808 nm laser light source at a fixed power density of 6.6 W cm^{-2}). The irradiation was performed on the BGNPs-biomolecular corona (0.25 mM Au^0). After irradiation, the samples were washed in water by centrifugation before TEM analysis.

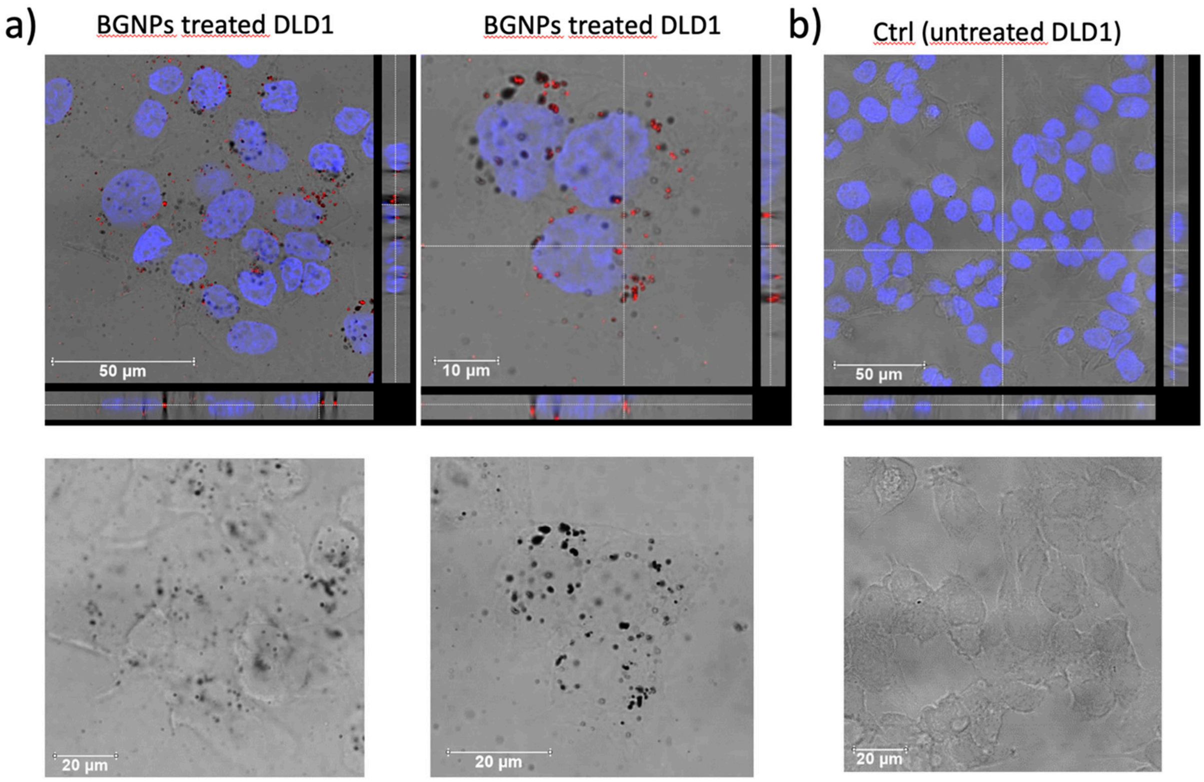

Figure S3. Confocal microscopy analysis of BGNPs uptake. Fluorescence (Hoechst staining), reflectance and transmission images overlapped (above) and transmission images (below) for **a**) BGNPs treated and **b**) untreated DLD1 cells. Cell nuclei are reported in blue and BGNPs in red.

References

1. Kuttner, C.; Höller, R.P.M.; Quintanilla, M.; Schnepf, M.J.; Dulle, M.; Fery, A.; Liz-Marzán, L.M. SERS and plasmonic heating efficiency from anisotropic core/satellite superstructures. *Nanoscale* **2019**, *11*, 17655–17663.
2. Quintanilla, M.; Kuttner, C.; Smith, J.D.; Seifert, A.; Skrabalak, S.E.; Liz-Marzán, L.M. Heat generation by branched Au/Pd nanocrystals: Influence of morphology and composition. *Nanoscale* **2019**, *11*, 19561–19570.
3. Roper, D.K.; Ahn, W.; Hoepfner, M. Microscale heat transfer transduced by surface plasmon resonant gold nanoparticles. *J. Phys. Chem. C* **2007**, *111*, 3636–3641.
